# Evaluation of lung volumes, vital capacity and respiratory muscle strength after cervical, thoracic and lumbar spinal surgery: Avaliação dos volumes pulmonares, capacidade vital e força dos músculos respiratórios no pós-operatório de cirurgia na coluna cervical, torácica e lombar

**DOI:** 10.1590/1516-3180.2014.00252601

**Published:** 2015-08-21

**Authors:** Marcio Aparecido Oliveira, Milena Carlos Vidotto, Oliver Augusto Nascimento, Renato Almeida, Ilka Lopes Santoro, Evandro Fornias Sperandio, José Roberto Jardim, Mariana Rodrigues Gazzotti

**Affiliations:** I PT, MSc. Researcher in the Neurosurgery/Respiratory Physiotherapy Group of the Respiratory Division, Universidade Federal de São Paulo (Unifesp), São Paulo, Brazil; II PT, PhD. Associate Professor of the Department of Physiotherapy, Universidade Federal de São Paulo (Unifesp), São Paulo, Brazil; III MD. Attending Physician in the Respiratory Division, Universidade Federal de São Paulo (Unifesp), São Paulo, Brazil; IV PT. Researcher in the Neurosurgery/Respiratory Physiotherapy Group of the Respiratory Division, Universidade Federal de São Paulo (Unifesp), São Paulo, Brazil; V MD. Attending Physician in the Respiratory Division, Universidade Federal de São Paulo (Unifesp), São Paulo, Brazil; VI PT, PhD. Researcher in the Neurosurgery/Respiratory Physiotherapy Group of the Respiratory Division, Universidade Federal de São Paulo (Unifesp), São Paulo, Brazil; VII MD. Assistant Professor in the Respiratory Division, Universidade Federal de São Paulo (Unifesp), and Director of the Pulmonary Rehabilitation Center, Unifesp, São Paulo, Brazil; VIII PT, PhD. Coordinator of the Neurosurgery/Respiratory Physiotherapy Group of the Respiratory Division, Universidade Federal de São Paulo (Unifesp), São Paulo, Brazil

**Keywords:** Respiratory function tests, Spine, Muscle strength, Postoperative period, Laminectomy, Testes de função respiratória, Coluna vertebral, Força muscular, Período pós-operatório, Laminectomia

## Abstract

**CONTEXT AND OBJECTIVE:**

Studies have shown that physiopathological changes to the respiratory system can occur following thoracic and abdominal surgery. Laminectomy is considered to be a peripheral surgical procedure, but it is possible that thoracic spinal surgery exerts a greater influence on lung function. The aim of this study was to evaluate the pulmonary volumes and maximum respiratory pressures of patients undergoing cervical, thoracic or lumbar spinal surgery.

**DESIGN AND SETTING:**

Prospective study in a tertiary-level university hospital.

**METHODS:**

Sixty-three patients undergoing laminectomy due to diagnoses of tumors or herniated discs were evaluated. Vital capacity, tidal volume, minute ventilation and maximum respiratory pressures were evaluated preoperatively and on the first and second postoperative days. Possible associations between the respiratory variables and the duration of the operation, surgical diagnosis and smoking status were investigated.

**RESULTS:**

Vital capacity and maximum inspiratory pressure presented reductions on the first postoperative day (20.9% and 91.6%, respectively) for thoracic surgery (P = 0.01), and maximum expiratory pressure showed reductions on the first postoperative day in cervical surgery patients (15.3%; P = 0.004). The incidence of pulmonary complications was 3.6%.

**CONCLUSIONS:**

There were reductions in vital capacity and maximum respiratory pressures during the postoperative period in patients undergoing laminectomy. Surgery in the thoracic region was associated with greater reductions in vital capacity and maximum inspiratory pressure, compared with cervical and lumbar surgery. Thus, surgical manipulation of the thoracic region appears to have more influence on pulmonary function and respiratory muscle action.

## Abstract (pt)

**CONTEXTO E OBJETIVO:**

Estudos têm demonstrado que alterações fisiopatológicas no sistema respiratório podem ocorrer após cirurgia torácica e abdominal. A laminectomia é considerada uma cirurgia periférica, mas é possível que as cirurgias de coluna torácica exerçam maior influência sobre a função pulmonar. O objetivo do estudo foi avaliar os volumes pulmonares e as pressões respiratórias máximas em pacientes submetidos a cirurgia de coluna cervical, torácica ou lombar.

**TIPO DE ESTUDO E LOCAL:**

Estudo prospectivo em hospital universitário terciário.

**MÉTODOS:**

Sessenta e três pacientes submetidos a laminectomia com diagnóstico tumor ou hérnia de disco foram avaliados. Foram avaliados, no pré-operatório, no primeiro e no segundo dias de pós-operatório, capacidade vital, volume corrente, volume por minuto e pressões respiratórias máximas. Possíveis associações entre as variáveis respiratórias e duração da cirurgia, diagnóstico cirúrgico e tabagismo foram investigadas.

**RESULTADOS:**

A capacidade vital e a pressão inspiratória máxima apresentaram redução no primeiro dia de pós-operatório (20.9% and 91.6%, respectivamente) nas cirurgias torácicas; P = 0,01), e a pressão expiratória máxima apresentou redução no primeiro dia de pós-operatório de cirurgia cervical (15.3%; P = 0,004). A incidência de complicações pulmonares foi de 3,6%.

**CONCLUSÕES:**

Houve redução da capacidade vital e das pressões respiratórias máximas no período pós-operatório em pacientes submetidos a laminectomia. A cirurgia na região torácica apresentou associação com maiores reduções na capacidade vital e na pressão inspiratória máxima em comparação com a cirurgia cervical e lombar. Assim, a manipulação cirúrgica da região torácica parece ter maior influência na função pulmonar e na ação dos músculos respiratórios.

## INTRODUCTION

Studies have shown that physiopathological changes to the respiratory system can occur following thoracic and abdominal surgery.^1^^-^ 4 The most frequently occurring changes are reduction in lung volume, changes to breathing patterns, altered gas exchange with reduction in partial arterial oxygen pressure (PaO2), increase in partial arterial carbon pressure (PaCO2) in the arterial blood and impairment of mucociliary transport.^5^^-^ 7 Recent studies have demonstrated that similar changes also occur in patients who undergo craniotomy, which is considered to be a peripheral surgical procedure. These patients experience reduction in lung volume, changes to respiratory patterns (from predominantly diaphragmatic to intercostal), hypoxemia and reduction in mucociliary transport.^8^^,^ 9 Other factors can contribute to the reduction in pulmonary volumes, such as general anesthesia, pain, immobility in bed and supine position.^5^^,^ 6 Up to 95% of patients with normal lungs who undergo general anesthesia may present atelectasis, which persists for more than 24 hours in 50% of the cases. Atelectasis is mainly present in dependent lung regions.

Laminectomy is considered to be a peripheral surgical procedure and is indicated for treating spinal column conditions.^10^ To the best of our knowledge, only one previous study has evaluated lung function in adult patients undergoing laminectomy to treat disc herniation or tumors.^11^ The changes to lung function and the behavior of the respiratory muscles were similar to those seen during the postoperative period following thoracoabdominal^4^ and cranial^8^^,^ 9 surgery. Alterations to respiratory function, such as reduction of lung volumes and respiratory muscle strength during the postoperative period following elective spinal surgery using a posterior access for tumor removal or herniated disc were correlated with surgical duration ≥ 240 minutes, presence of tumors, or cervicothoracic surgical access.^11^ However, these analyses were performed by pooling cervical surgery and thoracic surgery patients, due to the small number of patients in the cervical group.^11^ It is possible that thoracic spinal surgery exerts a greater influence on lung function, due to stimulation of reflexes that inhibit the action of the diaphragm.

## OBJECTIVE

The aim of this study was to evaluate how lung volume and maximum inspiratory and expiratory pressure behave in patients who have undergone spinal (cervical, thoracic, or lumbar) surgery for treatment of disc herniation or tumors.

## METHODS

This was a prospective study, conducted at a tertiary-level university hospital, on patients who underwent elective spinal surgery to correct a herniated disc or remove a tumor. The patients were divided into three groups, based on the location of the spinal surgery: cervical, thoracic or lumbar. This study received approval from the hospital's Human Research Ethics Committee (process 1956/07).

The inclusion criteria were that the patients needed to have undergone elective laminectomy under general anesthesia for disc herniation or tumor resection; to be ≥ 18 years of age; and to have signed the informed consent. The exclusion criteria were any presence of preoperative respiratory symptoms or obstructive or restrictive lung disease based on self-reports or clinical and radiological analysis; inability to perform spirometric or manometric procedures; postoperative respiratory support lasting more than 24 hours; pain greater than two points on the visual analog scale (VAS) during evaluations after the administration of analgesic medication; and death or brain death during the postoperative period.

Preoperative and postoperative evaluations were performed by the same investigator (MAO). The preoperative evaluation consisted of a clinical examination; investigation of past history of lung disease and smoking status; assessment of any presence of pain; and measurement of ventilation, vital capacity (VC) and respiratory muscle strength. The first and second-day postoperative follow-ups consisted of pain evaluation and measurement of ventilation, VC and maximum respiratory pressures. All the patients received training so that they would be able to carry out the maneuvers during the preoperative period.

Pain was measured using a VAS scale from 0 (no pain) to 10 (worst possible pain), which made use of cartoon faces to depict pain intensity.^12^ Based on pain intensity, the patients were initially medicated orally with an anti-inflammatory drug (tenoxicam), followed first by amitriptyline and then by carbamazepine and amplictil. A narcotic (tramadol) was administered in cases of intense pain. All measurements were performed with the patients in the dorsal decubitus position and experiencing little or no pain (≤ 2 on the pain scale). If pain persisted after treatment, the patient was excluded from the study, due to the possibility that their pain might influence the results. 

VC, tidal volume (TV) and minute ventilation (VE) were measured using a spirometer (Ohmeda RM 121, Tokyo, Japan), with the patient in the dorsal decubitus position and the bed positioned at zero degrees.

To measure VC, the patient was instructed to perform maximal slow expiration, starting from total lung capacity, through a mouthpiece connected to the spirometer. This procedure was repeated five times or until one measurement was lower than the previous one. The two highest measurements were not to differ by more than 5%, and the highest measurement was used for analysis. Measurements were expressed as predicted values.^13^ To measure VE, the patient was instructed to breathe normally for one minute through a facial mask connected to the spirometer. TV was determined by dividing VE by the respiratory rate (f).

Maximum inspiratory and expiratory pressures (MIP and MEP, respectively) were measured using a manometer (Comercial Médica, Brazil), using a nasal clip, with the patient in the dorsal decubitus position and the bed at zero degrees. To measure MIP, the patient performed maximal inspiratory effort at residual volume against a closed valve. To measure MEP, the patient performed maximal expiratory effort at total lung capacity against a closed valve. The measurements were repeated five times or until one measurement was lower than the previous one. The two highest measurements were not to differ by more than 5%, and the highest measurement was used for analysis. Measurements were compared using the predicted values.^14^ MIP and MEP were evaluated in order to measure the respiratory muscle strength.

The duration of the operation (in minutes) was obtained from the anesthesia file. The following were considered to be postoperative pulmonary complications: acute respiratory infection (pneumonia or purulent tracheobronchitis), atelectasis and/or bronchospasm. 

### Data analysis 

Analysis of variance (ANOVA) with repeated measurements was used to compare two or more dependent samples. Fisher's exact test or the chi-square test was used for categorical data. Continuous data were expressed as mean ± standard deviation. Statistical significance was set at 5% (P ≤ 0.05). The SPSS 17.0 software for Windows was used for the analyses.

The fpower macro of the SAS software, version 1.2 (Michael Friendly), was used to calculate the sample size, taking into consideration an ANOVA model for repeated measurements with the following factor groups: surgery type (cervical, lumbar or thoracic) and evaluation time (preoperative, first postoperative day and second postoperative day). Based on the findings reported by Di Pietro et al.^11^ and the preliminary results from the present investigation, the comparison of interest was based on VC. By taking the significance level to be 5% and the test power to be 80%, the minimum sample was determined to be 16 patients in each group.^15^

## RESULTS

Eighty-four patients fulfilled the inclusion criteria. However, twenty-one were excluded: eleven refused to participate; five were unable to understand the procedures; three had intense pain during the evaluation; one was on a ventilator for more than 24 hours; and one died. Among the remaining 63 patients, 20 underwent cervical surgery, 18 underwent thoracic surgery and 25 underwent lumbar surgery. Regarding tobacco use, 25.4% of the patients were smokers, 36.5% were ex-smokers and 38.1% had never smoked; no significant differences among the groups were found regarding smoking status (P = 0.73) or total number of pack-years (P = 0.95) (Table 1). Surgery to treat disc herniation was performed on 41 patients (65.1%) and to treat tumors on 22 patients (34.9%) (Table 1). The median duration of surgery was 240 minutes (mean: 236.7 ± 93.9 minutes). No significant difference in the duration of the operation was found among the three groups (P = 0.74) (Table 1).

**Table 1.** Patients' characteristics, diagnoses and duration of surgery according to location of surgery

	Location of surgery			P
	Cervical (n = 20)	Thoracic (n = 18)	Lumbar (n = 25)	
Age (years)	47.2 ± 15.6	45.1 ± 14.0	43.3 ± 10.3	0.618
Sex n (%)				
Male	17 (85%)	13 (72.2%)	13 (52%)	0.09
Female	3 (15%)	5 (27.8%)	12 (48%)	
Weight (kg)	74.4 ± 13.6	73.2 ± 14.3	72 ± 12.1	0.834
Height (cm)	170.6 ± 6.7	170.1 ± 10.0	167.6 ± 7.7	0.416
Body mass index (kg/m^2^)	25.6 ± 4.4	25 ± 3.0	25.6 ± 3.9	> 0.05
Smoking status				
Smoker	6 (37.5%)	5 (31.3%)	5 (31.3%)	0.732
Ex-smoker	5 (21.7%)	7 (30.4%)	11 (47.8%)	
Tobacco consumption (pack-years)	13 ± 20.2	14.5 ± 18.2	12.8 ± 18.4	0.952
Diagnosis				
Disc herniation	19 (95%)	1 (5.6%)	21 (84%)	< 0.001
Tumor	1 (5%)	17 (94.4%)	4 (16%)	
Duration of surgery (minutes)	223.8 ± 62.7	238.9 ± 90.2	245.6 ± 117.0	0.742
T ≤ 240 min	11 (55%)	7 (42%)	13 (52%)	0.232
T ≥ 240 min	9 (45%)	11 (58%)	12 (48%)	

Data are expressed as mean ± standard deviation or absolute and relative (%) frequency.

The incidence of pulmonary complications was 3.2%. Atelectasis occurred in one patient (5%) who underwent cervical laminectomy, and tracheobronchitis occurred in one patient (5.6%) who underwent thoracic surgery. One death related to hemodynamic instability occurred in the cervical group immediately following surgery. 

No differences in TV (P = 0.06) or VE (P = 0.34) were found among the groups during the postoperative period or within the groups at the different evaluation times. Cervical surgery did not result in any significant change in VC during the postoperative period, whereas thoracic surgery led to a 20.9% reduction in VC on the first postoperative day (P = 0.003) and a 17.4% reduction on the second postoperative day (P = 0.01). Lumbar surgery led to an 11% reduction in VC on the first postoperative day (P < 0.001). The reduction in VC was not associated with smoking status or the duration of the operation in any of the groups. The VC on the first and second postoperative days was higher in patients with a diagnosis of herniation than in those with tumors (P = 0.049 and P = 0.019, respectively). 

A 47% reduction in MIP occurred in the cervical group on the first postoperative day (P = 0.03), and this was maintained (43.7% reduction) on the second postoperative day (P = 0.01). In the thoracic group, the reductions were 91.6% and 89.2% on the first and second postoperative days, respectively (P < 0.001). In the lumbar group, the reductions were 55.7% and 53.4% on the first and second postoperative days, respectively (P < 0.001) (Table 2). No association was found between reduction in MIP and smoking status or surgical diagnosis. On the first postoperative day, MIP was 29.2% lower (P = 0.04) in patients whose surgery lasted more than 240 minutes than in patients with a duration of surgery of less than 240 minutes. No significant difference was found on the second postoperative day.

**Table 2.** Vital capacity, maximum inspiratory pressure and maximum expiratory pressure (% predicted) over time, among patients who underwent cervical, thoracic or lumbar laminectomy

	Preoperative	1^st^ PO day	2^nd^ PO day
Vital capacity			
Cervical	77.1 ± 16.7	64.4 ± 15.5	71.1 ± 17
Thoracic	79.4 ± 19.8	58.5 ± 15.4^*^	62 ± 17.5^*^
Lumbar	84.8 ± 8.6	73.8 ± 8.9^*^	79.1 ± 8.5
Maximum inspiratory pressure			
Cervical	122.5 ± 41.1	75.5 ± 22.1^*^	78.8 ± 19^*^
Thoracic	148.6 ± 40.9	57 ± 21^*^	59.4 ± 18.9^*^
Lumbar	128.7 ± 29.7	73.3 ± 21.1^*^	75.3 ± 21.5^*^
Maximum expiratory pressure			
Cervical	75.2 ± 17.6	59.9 ± 14.9^*^	67.7 ± 20
Thoracic	68.4 ± 19	52.8 ± 19	57.8 ± 18
Lumbar	86.2 ± 19	78.6 ± 20.8	80.3 ± 20

Data are expressed as mean ± standard deviation; PO = postoperative;

* compared with preoperative (P < 0.05).

A significant reduction (15.3%) in MEP occurred only in the cervical group in the first postoperative day (P = 0.02) (Table 2). No associations were found between reduction in MEP and smoking status or surgery duration. The MEP on the first and second postoperative days was higher in patients with diagnoses of tumors than in those with herniation (P = 0.024 and P = 0.046, respectively).

## DISCUSSION

In the present study, we observed that in patients who underwent spinal surgery to treat herniated discs or tumors, the greatest reduction in VC occurred in thoracic surgery patients with a tumor diagnosis. Inspiratory muscle strength was significantly reduced in all groups, with no significant association with the surgery site. However, the reduction was greater among patients with a surgical duration of over four hours. The reduction in expiratory muscle strength was greater in patients who underwent cervical or thoracic surgery than in those who underwent lumbar surgery. However, there was only a statistical difference in the group that underwent cervical surgery.

There has been increasing interest in the effects of surgical procedures on the respiratory system. Thoracic and upper abdominal surgical procedures are known to exert a negative influence on lung function and respiratory muscle strength.^16^^-^ 18 In upper abdominal surgery, the incidence of pulmonary complications ranges from 10% to 81%, whereas the risk of such complications in lower abdominal surgery is inversely proportional to the distance between the surgical incision and the umbilicus, and ranges from 0% to 5%. In peripheral surgery (i.e. outside the thoracoabdominal compartment), the incidence of complications is around 2%.^19^^,^ 20

The incidence of pulmonary complications in the present study was 3.6%, which was similar to the rate expected for lower abdominal surgery,^5^ lower than that of upper abdominal surgery^21^ and higher than that of peripheral surgery.^17^ Pulmonary complications have been correlated with anesthesia, supine positioning in the immediate postoperative period, immobility in bed or postoperative pain.^11^ However, in this study, pain was controlled with analgesics, and early mobilization was encouraged in order to reduce the possibility of respiratory complications. 

No association was found between changes in lung function and smoking status, although 61.5% of the participants were either smokers or ex-smokers. There is no consensus regarding the association between smoking status and decline in lung function during the postoperative period following upper abdominal or neurological surgery. Some studies have found an association between smoking status and the incidence of pulmonary complications,^20^ while others have not found this correlation.^22^^,^ 23

Reductions in VC and respiratory muscle strength occurred in all groups in our study, with a greater negative impact (diminished VC) on the thoracic group than on the lumbar group. Several studies have reported pulmonary changes in neurosurgery patients that potentially increased the risk of complications.^8^^,^ 9 ^,^ 11 Di Pietro et al.^11^ were unable to detect changes in lung function or respiratory strength stemming from surgery in the cervical and thoracic regions, due to their small sample size. However, the anatomical and physiological differences between these regions might trigger different responses from the respiratory system. 

In the present study, a 20.9% reduction in VC was found on the first postoperative day in patients who underwent thoracic surgery (P = 0.003). This was similar to the 25% reduction over the same timeframe following craniotomy that was found by Sogame et al.^22^ The reduction in VC following a craniotomy procedure that these authors found was similar to what had been described in lower abdominal surgery, and they attributed this reduction to the anesthetic effect on the respiratory system, since no thoracoabdominal manipulation had been performed. 

A 29.2% reduction in MIP was found on the first postoperative day (P = 0.041) in patients whose duration of surgery was ≥ 240 minutes, in comparison with those whose surgery was < 240 minutes, with no significant differences between the different groups studied. Sogame et al.^22^ found that duration of surgery ≥ 240 minutes was associated with a reduction in both VC and TV on the first postoperative day, in patients who underwent elective craniotomy to correct aneurysm. Di Pietro et al.^11^ found that reductions in VC, TV, MIP and MEP were also associated with duration of surgery ≥ 240 minutes. The reduction in inspiratory muscle strength suggests that lengthy surgery accentuates the inhibition of the diaphragm reflex, independent of the location of the surgery.^8^^,^ 9 ^,^ 23

Reduction in respiratory muscle strength is related to diminished lung function and an increase in the incidence of pulmonary complications in patients who undergo surgery.^24^ The overall reductions in MIP of 52.8% and 51.7% on the first and second postoperative days, respectively (P < 0.001), were similar to the MIP reduction found following upper abdominal surgery,^24^^,^ 25 and much higher than the 18% reduction in MIP expected for laminectomy of the lumbar spine.^12^ It has been recognized that inhibition of the diaphragm reflex is the main factor for lung volume reduction following thoracic or abdominal surgery, even if direct manipulation of the thoracoabdominal viscera does not take place.^26^^,^ 27 It is possible that laminectomy may cause inhibition of this reflex, thereby leading to reductions in lung volume and respiratory muscle strength, as found in the present study. The reductions in lung volume and respiratory muscle strength increase the risk of postoperative complications due to atelectasis, pneumonia, respiratory failure and exacerbation of underlying chronic lung disease.

Reduced respiratory measurements and respiratory muscle strength may be related to pain, fear or lack of cooperation from the patient.^24^ However, pain greater than two points on the visual analog scale (VAS) during evaluations after administration of analgesic medication was an exclusion criterion in the present study, which suggests that this factor did not exert an influence on our findings. Thus, it seems that our findings were, in fact, associated with inhibition of the diaphragm reflex caused by the surgical procedure. 

This study has some limitations that should be noted. Although the pain was controlled, fear and precaution in performing maximal respiratory strength evaluations during the postoperative period may have had some influence on the measurements. However, we believe that this is unlikely to have changed our results significantly, since the patients were well-oriented regarding pain control through medication. Moreover, they had undergone training to carry out the maneuvers during the preoperative period. Other limitations on this study are that it was conducted in a single center and that the sample size was relatively small. 

The study ended after the second postoperative day because most of the patients were discharged after the second day. The patients were informed by the physiotherapy team about the changes to the respiratory system inherent to anesthesia and the surgical procedure, but no physiotherapeutic intervention was carried out. Our findings of reductions in VC and respiratory muscle strength justify differentiated, prophylactic intervention by the entire multidisciplinary team. 

## CONCLUSIONS

Based on our findings, thoracic laminectomy appears to exert a greater negative influence on lung function and respiratory muscle strength during the postoperative periods, in comparison with cervical and lumbar laminectomy.
